# rRNA synthesis inhibitor, CX-5461, activates ATM/ATR pathway in acute lymphoblastic leukemia, arrests cells in G2 phase and induces apoptosis

**DOI:** 10.18632/oncotarget.4093

**Published:** 2015-06-05

**Authors:** Sandeep S. Negi, Patrick Brown

**Affiliations:** ^1^ Department of Oncology and Pediatrics, Johns Hopkins University School of Medicine, Baltimore MD, USA

**Keywords:** CX-5461, rRNA synthesis, ATM/ATR pathway, G2/M arrest, acute lymphoblastic leukemia

## Abstract

Ribosome biogenesis is a fundamental cellular process and is elevated in cancer cells. As one of the most energy consuming cellular processes, it is highly regulated by signaling pathways in response to changing cellular conditions. Many of the regulators of this process are aberrantly activated in various cancers. Recently two novel rRNA synthesis inhibitors, CX-5461 and BMH-21, have been shown to selectively kill cancer cells while sparing normal cells. Here, we tested the effectiveness of pre-rRNA synthesis inhibitor CX-5461 on acute lymphoblastic leukemia cells with different cytogenetic abnormalities. Acute lymphoblastic leukemia cells are more sensitive to rRNA synthesis inhibition compared to normal bone marrow cells. CX-5461 treated cells undergo caspase-dependent apoptosis independent of their p53 status. More-over, CX5461, activates checkpoint kinases and arrests cells in G2 phase of cell cycle. Finally, overcoming this G2 arrest by inhibiting ATR kinase leads to robust cell killing. These results show that CX-5461 can be even more potent in combination with ATR inhibitors.

## INTRODUCTION

Nucleolus is the site of rRNA synthesis and ribosomal subunit assembly [1]. It is a dynamic structure and many of its constituents constantly exchange with the nucleoplasm during interphase [2]. As one of the most energy intensive processes, ribosome biogenesis is constantly fine-tuned in response to growth conditions, cellular stress and cell cycle. As a structure formed to provide efficient ribosome biogenesis, nucleolus disassembles at the onset of mitosis and reassembles during telophase, mirroring the inhibition of rRNA synthesis during prophase and its activation during telophase [3]. Similarly, nucleolus also unravels in response to inhibition of ribosome biogenesis by certain drugs like 5-FU, Actinomycin D and DRB [4]. Apart from its conventional role in ribosome biogenesis, its non-traditional functions include sensing cellular stress and control of aging [3, 5, 6]. Changes in nucleolar morphology and functions are widely observed in cancer tissues [7]. Many molecular changes that drive various cancers have been shown to modulate rRNA synthesis. For example, AKT activation enhances rRNA synthesis and promotes tumor growth, and both B and T-acute lymphoblastic leukemia cells are very sensitive to AKT inhibition [8, 9]. c-Myc, which is over-expressed in a variety of hematological malignancies, associates with ribosomal DNA and activates RNA polymerase I transcription [10, 11, 12]. The AML1-ETO fusion protein epigenetically controls cell growth through up-regulation of rRNA synthesis in acute myelogenous leukemia (AML) cells [13].

CX-5461 and BMH-21, two recently developed rRNA synthesis inhibitors, have been shown to have therapeutic effects on a wide range of cancer cell lines, with the most significant effects seen with hematological cancers [14–15]. More importantly, they have been shown to be selectively cytotoxic to cancer cells with minimal effect on normal cells. BMH-21 was discovered in a small molecule library screen for p53 activating compounds with antitumor activity [16]. It binds to GC rich region, present at high frequency in rDNA region, and inhibits RNA Pol I transcription independent of DNA damage response [15, 17]. Interestingly, BMH-21 antitumor activity is associated with proteasome dependent degradation of a catalytic subunit of RNA Pol I complex. CX-5461 was first described by Drygin et al. [14] as a novel small molecule inhibitor specific for RNA Pol I multi-protein complex. It was discovered in a chemical screen for compounds that selectively inhibit RNA pol I transcription relative to Pol II transcription. Similar to BMH-21, it selectively induces cell death in cancer cells but has a different mechanism of action. It inhibits the interaction between SL1 and rDNA thereby preventing the formation of pre-initiation complex. Bywater et al. [18] showed that CX-5461 disrupts nucleolar structure and its therapeutic effect is p53-dependent. CX-5461 is currently in a phase 1 clinical trial for hematological malignancies and has been shown to be effective in mouse models of B-lymphoma and MLL-AF9 AML [18].

As the cell lines used in previous study included only one acute lymphoblastic leukemia (ALL) cell line, we tested the effectiveness of pre-rRNA synthesis inhibitor on acute lymphoblastic leukemia cells with different cytogenetic abnormalities. We further investigated the effect of rRNA synthesis inhibition on cell cycle distribution. We showed that ATM/ATR pathway is activated by CX-5461 treatment resulting in G2 phase arrest. Finally, we showed that inhibition of ATR pathway activation enhances CX-5461 mediated apoptosis.

## RESULTS

### CX-5461 inhibits proliferation of ALL cells

**Figure 1:**
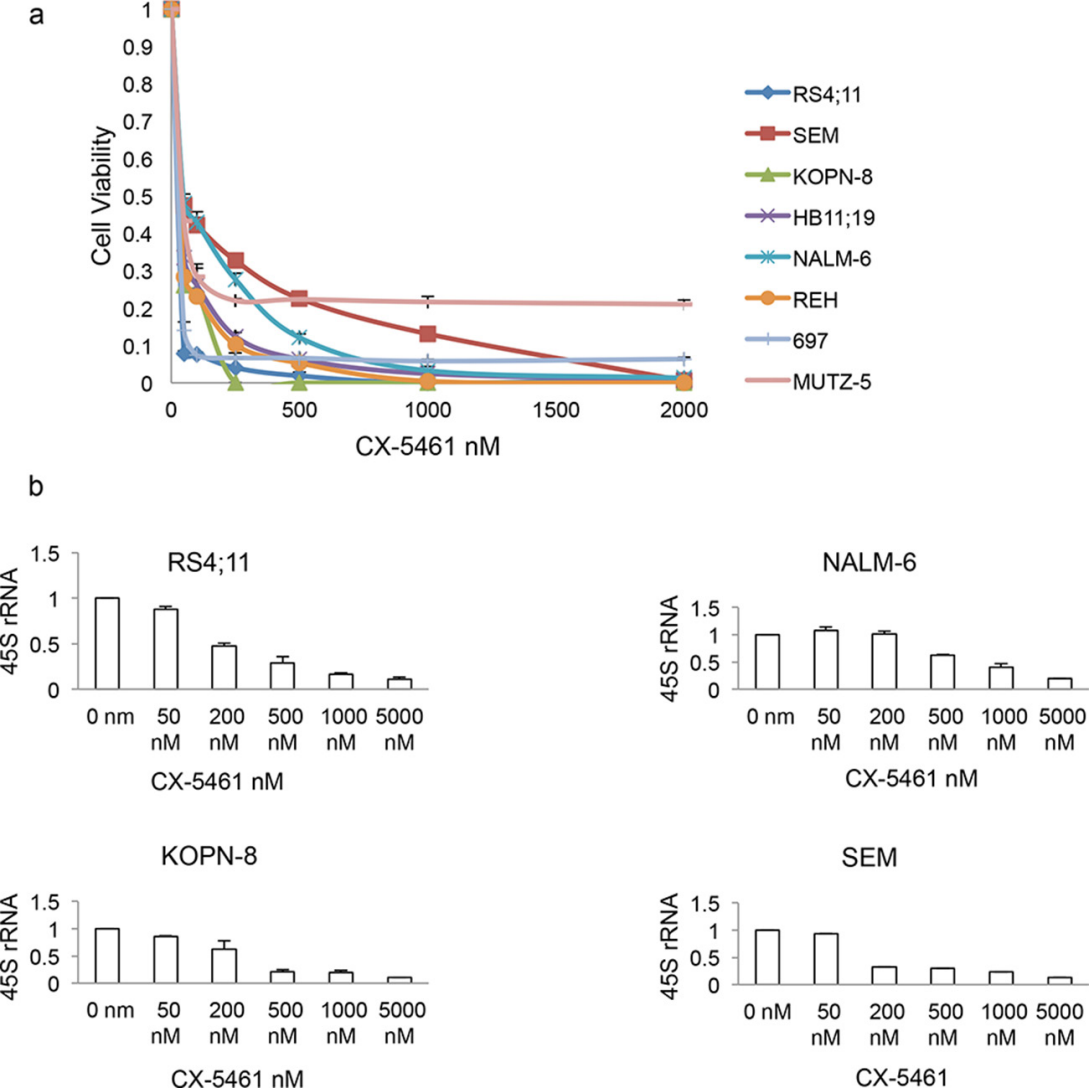
CX-5461 inhibits growth in acute lymphoblastic leukemia (ALL) cells **a.** All eight ALL cell lines showed marked decrease in proliferation after a 3 day treatment with CX-5461. **b.** 3 h treatment with CX-5461 reduced 45S pre-rRNA transcript in a dose dependent manner. Transcript levels were measured using quantitative PCR and normalized to the expression of GAPDH and Actin. (a, b) Experiments were repeated three times and error bars represent +/− S.D.

CX-5461 has previously shown anti-proliferative activity in many solid cancer lines of NCI-60 panel. As that panel had only one acute lymphoblastic leukemia cell line, we tested the therapeutic potential of CX-5461 on a range of ALL cell lines. We treated 8 ALL cell lines with varied cytogenetic abnormalities with increasing concentrations of CX-5461 for 3 days (Supplementary Table 1). The drug showed robust inhibition of cell proliferation in the low nano-molar range in all cell lines tested (Fig. 1A). As CX-5461 block the formation of RNA Pol I pre-initiation complex, we investigated the pre-rRNA levels in CX-5461 treated cells lines. We choose 4 cell lines, SEM, KOPN-8, RS4;11 and NALM-6, to check the rRNA synthesis inhibition after drug treatment by qRT-PCR. As 45S pre-RNA has a very short half-life (10 min), its level in the cell is indicative of the rate of rRNA synthesis. We treated cells for 3 h with increasing concentration of CX-5461. All cell lines showed concentration dependent decrease in 45S pre-rRNA transcript (Fig. 1B).

### CX-5461 induces caspase-dependent apoptosis in ALL cells

**Figure 2:**
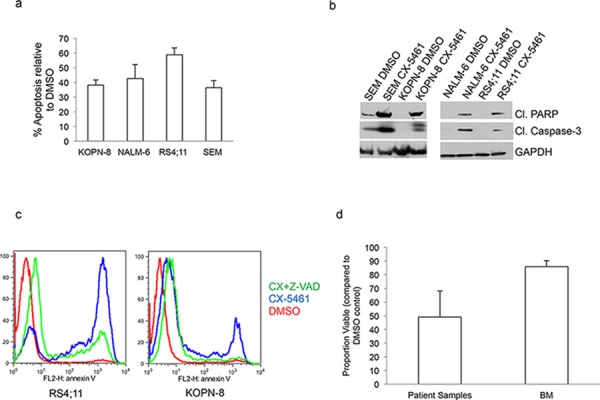
CX-5461 induces caspase dependent apoptosis in ALL cells **a.** Annexin V was used to measure apoptosis in ALL cell lines.% apoptosis relative to DMSO treated control is plotted. Histograms show the values (mean ± S.D.) of three independent experiments. **b.** Cells were treated with 0.25 μM CX-5461 for 1 day. Cleaved caspase-3, cleaved PARP and GAPDH antibodies were used for western blot. **c.** RS4;11 and KOPN-8 cells were pre-treated with pan-caspase inhibitor Z-VAD-FMK followed by 2 days of treatment with 0.5 μM CX-5461. Annexin V was used to measure apoptosis. One of two representative experiments is shown. **d.** Patient samples (*n* = 6) or normal bone marrow (*n* = 3) were treated with 1 μM CX-5461 or DMSO for 2 days and apoptosis was measured with Annexin V staining. Viable proportion is plotted normalized to DMSO treated samples. Results are shown as mean +/− S.D.

We next investigated if CX-5461 induced inhibition of proliferation is due to cell death. We treated SEM, KOPN-8, RS4;11 and NALM-6 cells with 0.25 μM CX-5461 or DMSO control and measured the induction of apoptosis by Annexin V staining. CX-5461 induced apoptosis in all four ALL cell lines compared to their respective DMSO treated controls (Fig. 2A). Further, western blot analysis showed increased levels of cleaved caspase-3 and cleaved PARP in CX-5461 treated ALL cell lines (Fig. 2B). To check if CX-5461 induced apoptosis is dependent on caspases, we used pan-caspase inhibitor Z-VAD-FMK. Pre-treatment with Z-VAD-FMK significantly reduced annexin V staining in CX-5461 treated cells confirming caspase-dependent apoptosis (Fig. 2C). We then tested the effectiveness of CX-5461 on ALL patient samples with different cytogenetic translocations. Six ALL patient samples with varied cytogenetic abnormalities (Supplementary Table 2) were treated with DMSO or 1 μM CX-5461 for 48 h and analyzed for the induction of apoptosis using Annexin V staining (Fig. 2D). The drug treated samples showed increased apoptosis compared to DMSO treated patient samples. All but one (MLL-AF4) CX-5461 treated sample show less than 50% viability compared to their DMSO treated control. We then checked for a therapeutic window for the drug. We treated bone marrow from three healthy individuals with 1 μM CX-5461 for 2 days (Fig. 2D). Normal cells showed minimal cell death at this concentration. This shows that there is a therapeutic window for treatment with CX-5461 without appreciable toxicity to healthy cells.

### CX-5461 induced apoptosis is p53 independent

**Figure 3:**
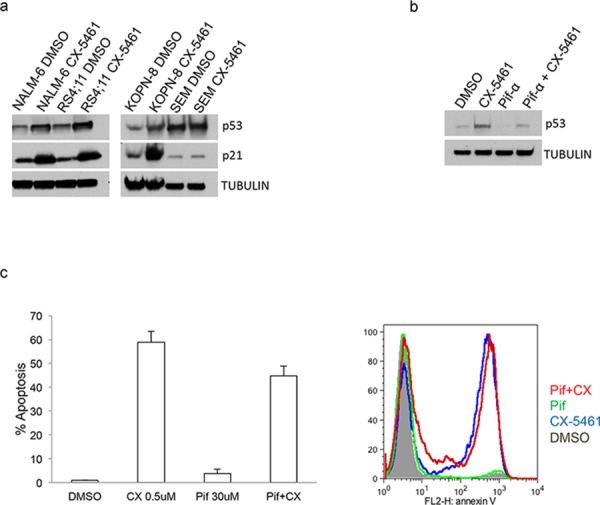
CX-5461 mediated apoptosis is p53 independent **a.** Two p53 wild type (NALM-6 and RS4;11) and two mutant (SEM and KOPN-8) cell lines were used. Expression of p53 and its downstream target p21 was shown with western blot upon 1 day treatment with 0.25 μM CX-5461. **b, c.** p53 wild type RS4;11 cells were treated with 0.25 μM CX-5461 or 30 μM of p53 inhibitor pifithrin-α or both. Western blot was used to measure p53 levels after 1 day drug treatment (b). Annexin V was used to measure apoptosis after 2 days. Histogram and representative flow cytometry data is shown (c). Experiments are repeated three times and plotted as mean +/− S.D.

Inhibition of rRNA synthesis has been shown to cause nucleolar stress that leads to p53 stabilization and p53-dependent apoptosis [19, 20]. An earlier report showed that p53 wild-type melanoma cell line A375 showed only modest induction of p53 upon treatment with CX-5461, even at a concentration 10 fold higher than its IC50 for RNA Pol I inhibition and viability [14]. That report did not find any correlation between p53 status and CX-5461 sensitivity in solid cancer cell lines. In hematological malignancies, however, there was a suggestion that p53 wild-type cells are more sensitive to CX-5461 treatment than mutant p53 cells. A subsequent report showed that p53 wild-type B-lymphomas are at least 2 orders of magnitude more sensitive to CX-5461 than p53 mutant B-lymphoma cells, and apoptosis in these cells is p53-dependent [18]. To check if CX-5461-induced apoptosis is p53-dependent in ALL, we treated two p53 wild-type (RS4;11 and NALM-6) and two p53 mutant cell lines (SEM and KOPN-8) with CX-5461 (Cosmic database and IARC p53 mutation database). Three of these cell lines (RS4;11, NALM-6, KOPN-8) showed significant increase in expression of p53 and p21 (a p53 downstream target gene), whereas SEM cells showed minimal increase in p53 or p21 expression upon CX-5461 treatment (Fig. 3A). We further confirmed p53 independent effect of CX-5461 on cellular apoptosis using p53 inhibitor pifithrin-α. As shown in Fig. 3B, pre-treatment of p53 wild-type cell line RS4;11 with pifithrin-α substantially reduced p53 activation upon CX-5461 treatment (Fig. 3B). However, this reduced p53 activation had only a modest effect on CX-5461 mediated apoptosis (Fig. 3C). Similar result was seen with another p53 wild type cell line NALM-6 (Supplementary Fig. 1). This suggests that p53-independent pathways are more dominant in CX-5461 mediated apoptosis in ALL.

### CX-5461 arrests cells in G2-phase of cell cycle

**Figure 4:**
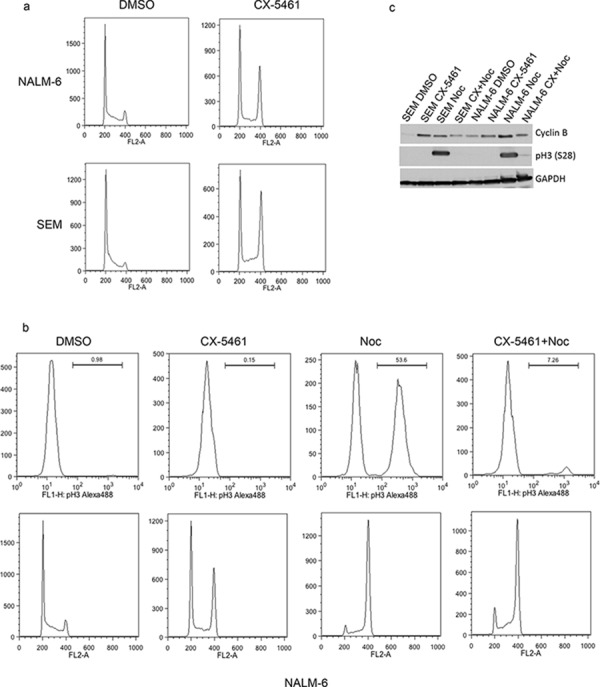
CX-5461 arrests ALL cells in G2 phase **a.** Cells were treated with 0.25 μM CX-5461 for 1 day. Cell-cycle distribution was determined by flow cytometry analysis of propidium iodide (PI) stained cells. One representative experiment out of three is shown. **b.** and **c.** NALM-6 and SEM cell were treated with CX-5461, Nocodazole or 2 h pre-treatment with CX-5461 followed by nocodazole for 1 day. Cell-cycle profiles were analyzed by flow cytometry using pH3(S28) as an indicator of mitosis (top panel) and PI for DNA content (bottom panel). (c) FACS results were confirmed with western blot by analyzing cyclin B and pH3(S28) levels.

To further understand the mechanism responsible for CX-5461 induced anti-proliferative effects, we analyzed the cell-cycle profile of CX-5461 treated ALL cells. We treated one p53 mutant (SEM) and one p53 wild type (NALM-6) cell lines with CX-5461 for one day and analyzed the cell-cycle distribution. Both cell lines showed G2/M phase arrest as shown by cell cycle distribution of propidium iodide stained cells (Fig. 4A). To differentiate between G2 and M phase arrest, we looked at mitosis specific histone H3 phosphorylation in these cells. Histone H3 is phosphorylated at multiple sites at the onset of mitosis (Ser 10 and 28) and is used as an M phase marker [21]. We used nocodazole which arrests cells in metaphase stage of mitosis as a control. Cell cycle analysis showed G2/M phase arrest of cells treated with only CX-5461, only nocodazole or both (Fig. 4A). As expected, cells treated with nocodazole showed marked increase in pH3(S28) signal (Fig. 4B). However, CX-5461 treated cells showed no increase in pH3 (S28) (Fig. 4B). Similar results were obtained with pH3(S10) antibody in SEM cells (Supplementary Fig. 2). We further confirmed these results by checking the levels of cyclin B, pCDC2(Y15) and pH3(S28) with western blot (Fig. 4C, Supplementary Fig. 3). Cyclin B expression varies during cell-cycle with the highest expression during G2/M phase. While cyclin B was high in both CX-5461 and nocodazole treated cells, pH3(S28) was only detected in nocodazole treated cells (Fig. 4C). Moreover, 3 h CX-5461 pre-treatment followed by nocodazole for 24 h did not lead to any increase in pH3(S28) suggesting that CX-5461 treatment activates the cellular machinery that inhibits their entry into M phase (Fig. 4B, 4C).

### CX-5461 activates ATM/ATR pathway

**Figure 5:**
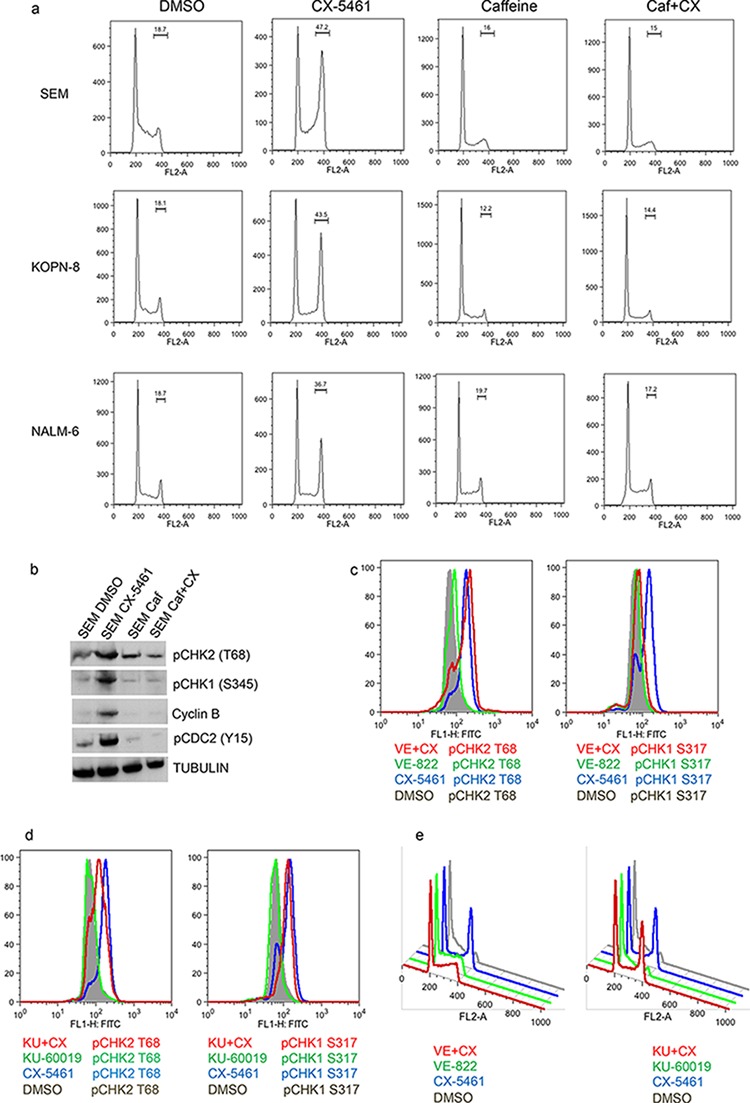
CX-5461 activate ATM/ATR pathway **a.** and **b.** SEM cells were treated with 0.25 μM CX-5461 or 1.5 mM caffeine alone or pre-treated with caffeine for 1 h followed by CX-5461 for 1 day. (a) Cell-cycle was analyzed by flow cytometry as before. Caffeine completely removed G2 block induced by CX-5461. (b) Western blot was used to measure the levels of proteins involved in G2/M checkpoint. **c-e.** SEM cells were pre-treated with 0.1 μM VE-822 (c) or 0.25 μM KU-60019 (d) for 1 h followed by 0.25 μM CX-5461 for 1 day. Levels of pCHK1(S317) and pCHK2(T68) (c and d) and cell cycle distribution (e) were measured by flow cytometry as before. One out of two representative experiments is shown.

To explore the mechanism of CX-5461 mediated G2 arrest, we checked for the involvement of checkpoint kinases. Ataxia telangiectasia-mutated (ATM) and ATM-Rad3-related (ATR) are responsible for the activation of checkpoint kinases CHK1 and CHK2 in response to cellular stress [22]. These checkpoint kinases induce G2 arrest in response to cellular stress by maintaining the inhibitory CDC2(Y15) phosphorylation that prevents entry into M phase. To test the involvement of ATM/ATR in CX-5461 mediated G2 arrest, we pre-treated cells with ATM/ATR inhibitor caffeine [23]. As shown in Fig. 5A, pre-treatment with caffeine completely abolished CX-5461 mediated G2 arrest. Western blot analysis of SEM cells show that CX-5461 increased pCHK1 and pCHK2 levels as wells as pCDC2 (Y15), indicating the activation of ATM/ATR pathway upon inhibition of rRNA synthesis (Fig. 5B). Interestingly, caffeine pre-treatment reduced cyclin B levels, reduced activation of checkpoint kinase CHK1 and CHK2 as wells as pCDC2 levels. We then used specific ATR and ATM inhibitor VE-822 and KU-60019, respectively, to decipher the relative contribution of these kinases in this G2 phase arrest. VE-822 has an IC50 of 19 nM and 2.6 μM for ATR and ATM inhibition respectively while KU-60019 has an IC50 of 6 nM and > 10 μM for ATM and ATR respectively [24, 25]. pCHK1(S317) and pCHK2(T68) are used as a measure of inhibition of ATR and ATM kinase activity. We treated SEM cells with 100 nM VE-822 and 250 nM KU-60019 alone and in combination with 250 nM CX-5461 and measured the levels of pCHK1 and pCHK2 by flow cytometry (Fig. 5C, 5D). VE-822 substantially reduced pCHK1 but showed no effect on pCHK2 levels induced by CX-5461 treatment. Similarly KU-60019 reduced the levels of pCHK2 with only a modest effect on pCHK1 levels. We then investigated if these specific ATM/ATR inhibitors will abrogate CX-5461 induced G2 arrest. Pre-treatment with ATR inhibitor VE-822 relieved CX-5461-induced G2 arrest whereas ATM inhibitor KU-60019 had no effect on G2 inhibition (Fig. 5E).

### ATR inhibitor enhances CX-5461 mediated apoptosis

**Figure 6:**
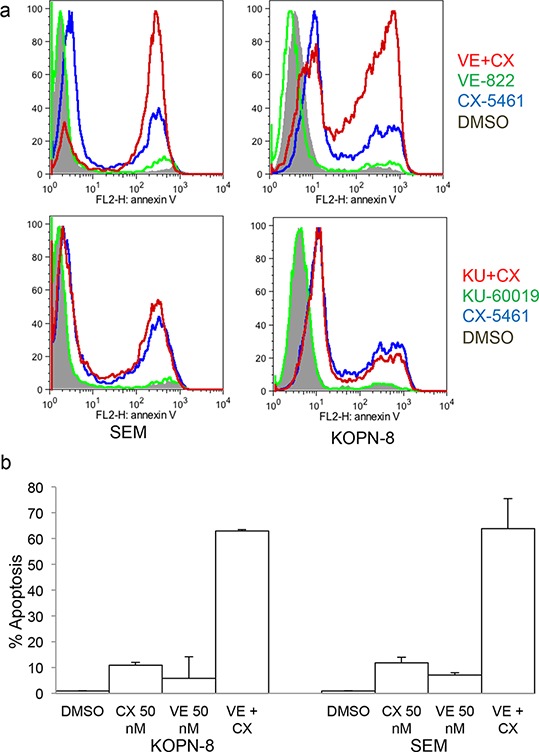
CX-5461 shows synergy with ATR inhibitor **a.** SEM and KOPN-8 were treated as in (Figure 5C) for 2 days and apoptosis was measured with Annexin V staining. **b.** SEM and KOPN-8 cells were pre-treated with 0.05 μM VE-822 followed by 0.05 μM CX-5461 or with CX-5461 or VE-822 alone for 2 days. Annexin V was used to measure apoptosis. (a, b) All experiments were repeated three times and error bars represent +/− S.D.

ATM/ATR inhibitors are shown to be effective in combination with radiotherapy/chemotherapy in inducing cell death. As G2 arrest allows cells to recover from the cellular stress, we tested ATM and ATR inhibitors in combination with CX-5461 to check if they will lead to more robust cell killing. Both the inhibitors showed minimal cell killing when used alone. While KU-60019 in combination with CX-5461 did not show any appreciable change in cell killing compared to CX-5461 alone, the combination of VE-822 and CX-5461 showed a marked increase in apoptosis compared to CX-5461 alone (Fig. 6A). We then tested if ATR inhibitor can sensitize these cells to a lower dose of CX-5461. We treated cells with a lower dose of VE-822 and CX-5461 that showed minimal cell death. At 50 nM, VE-822 and CX-5461 showed minimal induction of apoptosis when used alone but their combination greatly increased cell killing (Fig. 6B).

## DISCUSSION

Nucleolus is the most prominent sub-nuclear structure and the site of ribosome production in the cell. Many chemotherapeutic drugs used currently like actinomycin D, doxorubicin, camptothecin and 5-fluorouracil disrupt ribosome biogenesis. Burger et al. [26] suggested that inhibition of ribosome biogenesis may contribute to the efficacy of these drugs. Until recently it was difficult to conclude that ribosome biogenesis is a bona fide target for cancer therapy as these drugs are not selective for inhibition of rRNA synthesis alone. With the discovery of selective rRNA synthesis inhibitors, CX-5461 and BMH-21, nucleolus is again at the forefront of novel cancer targets [14, 15, 18].

Multiple studies have shown that inhibition of RNA Pol I transcription by inactivation of components of pre-initiation complex or by low dose actinomycin D cause nucleolar stress and disintegration [4, 19]. Nucleolar components are dispersed in nucleoplasm leading to p53 stabilization and cell-cycle arrest. Knockdown of *POLR1A* gene, the catalytic subunit of RNA Pol I, downregulates E2F-1 expression and accumulate cells in G1 phase [27]. Similarly, deletion of the transcription initiation factor 1A (*TIF-1A*), a RNA Pol I specific coactivator, leads to G1 arrest [19]. In case the cells are unable to overcome this stress, it leads to apoptosis. Our results also support early changes in cell-cycle modulators upon inhibition of rRNA synthesis as two hour pre-treatment with CX-5461 was enough to inhibit entry into mitosis in presence of nocodazole. More-over, overcoming G2 arrest with ATR inhibitor VE-822 led to enhanced apoptosis.

Nucleolus is a dynamic organelle and many of its constituents shuttle between the nucleolus and nucleoplasm. Many of the proteins are sequestered in the nucleolus and are released only in response to certain stimuli [28, 29]. It is possible that untimely release of these nucleolar components by CX-5461 treatment results in G2 arrest and apoptosis. rRNA transcription is cell cycle regulated being highest during late G1 to G2 phase and sharply decrease as the cell prepares to enter mitosis [30, 31]. This mirrors nucleolar assembly and disassembly as nucleolus is disassembled at the onset of mitosis and start to reassemble during telophase. It would be interesting to see if CX-5461 will show cell-cycle dependent cytotoxicity correlated with the rate of rRNA synthesis or will CX-5461 be effective in cells arrested in mitosis as rRNA synthesis is suppressed during mitosis. This will be especially important in a scenario where CX-5461 is used in combination with mitotic inhibitors like taxanes or vinca alkaloids. In this context, Wu et al. [32] showed that late S and G2 phase cells are most sensitive to actinomycin D treatment while mitotic cells were resistant.

In summary, we have shown that ALL cells are more sensitive to rRNA synthesis inhibition compared to normal bone marrow cells. This sensitivity is independent of their p53 status. More-over, CX-5461 activates ATM/ATR pathway leading to G2 phase arrest in these cells. This cell-cycle arrest provides time for cells to recover from drug induced stress and overcoming this G2 arrest by inhibiting ATR kinase leads to robust cell killing. Finally, ATR inhibitor can sensitize ALL cells to a lower CX-5461dose thereby potentially reducing its side effects.

## MATERIALS AND METHODS

### Cell lines and patient samples

RS4;11, REH, MUTZ-5, 697, SEM, KOPN-8 and NALM-6 cell lines were purchased from German Collection of Microorganisms and Cell Cultures (DSMZ). Informed consent was obtained in accordance with the institutional review board guidelines for the patient samples and normal bone marrow samples used in this study. Patient samples were layered on a Ficoll-Hypaque density gradient centrifugation and enriched blasts were stored in liquid nitrogen until further use. The diagnosis of ALL was based on morphology and flow cytometric analysis on immunophenotype. Cytogenetic was determined by standard procedures. Cell lines and patient samples used in this study are listed in supplementary table 1 and 2. CX-5461 was purchased from Xcess Biosciences; VE-822 and KU-60019 from Selleck Chemicals; Caffeine and Nocodazole from Sigma-Aldrich.

### Flow cytometry

Cells were pre-treated with caffeine, VE-822 or KU-60019 for 1 h followed by CX-5461 treatment. For nocodazole experiment, cells were pre-treated with CX-5461 for 2 h followed by nocodazole treatment. After drug treatments, cells were fixed in methanol and stored at −20°C till further processing. For cell-cycle analysis cells were spin down, washed twice in PBS and suspended in RNaseA containing propidium iodide (PI) solution and incubated for half an hour. Cells were run on BD FACScaliber flow cytometer (BD Biosciences) and final cell-cycle analysis was performed using FlowJo software (Tree Star). For phospho protein detection, fixed cells were incubated with pH3(S10)-FITC (Biolegend), pH3(S28) (Cell Signaling Technology), pCHK1(S317) (Cell Signaling Technology) or pCHK2(T68) (Cell Signaling Technology) and analyzed with flow cytometry.

### Western blot

Cell lysates were fractionated on SDS-PAGE gel and transferred to PVDF membrane. Membrane was blocked with 5% milk and incubated with primary antibody. Antibodies used were cleaved caspase-3 (Cell Signaling Technology), cleaved PARP (Cell Signaling Technology), p21(Cell Signaling Technology), p53 (BioLegend), GAPDH (Cell Signaling Technology), β-TUBULIN (Cell Signaling Technology), pCDC2(Y15) (Genewiz), Cyclin-B (Cell Signaling Technology), pCHK1(S317) (Cell Signaling Technology), pCHK1(S345) (Cell Signaling Technology), pCHK2(T68) (Cell Signaling Technology), pH3(S10) (Cell Signaling Technology), pH3(S28) (Cell Signaling Technology).

### Cell proliferation and apoptosis

Cells were seed in 96 well plates and incubated in DMSO (control) or different concentration of CX-5461 for 3 days. CellTiter 96 AQ_ueous_ One Solution Cell Proliferation solution (Promega) was added to each well and incubated for 1 h at 37°C in dark. Absorbance was recorded at 490 nm using Bio-Rad microplate reader. Results were background subtracted and normalized to DMSO treated control. Experiment was repeated three times and results were plotted as mean +/− S.D.

Annexin V was used for measuring apoptosis (BD Biosciences). Patient samples or cell lines were seeded in 6 well plates and incubated with DMSO or CX-5461. After 48 to 72 h cells were harvested, washed in PBS and suspended in Annexin V binding buffer. Annexin V was added to each sample and incubated in dark for 30 min. Cells were analyzed on BD FACScaliber flow cytometer. Results were normalized to control and plotted as mean +/− S.D. of three separate experiments.

### qPCR

Total RNA was extracted from cultured cells using RNeasy mini kit (Qiagen). One microgram of total RNA was reverse transcribed. qPCR was performed using SYBR Green mastermix and run on a CFX96 Bio-Rad real time PCR machine. Primer sequences for 45S pre-rRNA are forward 5′-CCGCGCTCTACCTTACCTACCT-3′ and reverse 5′-GCATGGCTTAATCTTTGAGACAAG-3′; for βActin are forward 5′-CGTCACCAACTGGGACGACA-3′ and reverse 5′-CTTCTCGCGGTTGGCCTTGG-3′. Experiments were repeated three times. Results were normalized to GAPDH and βACTIN expression for each sample and plotted as relative to the expression of control DMSO treated samples.

## SUPPLEMENTARY FIGURES AND TABLES



## References

[1] Olson MOJ (2011). The Nucleolus.

[2] Olson MO, Dundr M (2005). The moving parts of the nucleolus. Histochem Cell Biol.

[3] Dundr M, Misteli T, Olson MO (2000). The dynamics of postmitotic reassembly of the nucleolus. J Cell Biol.

[4] Rubbi CP, Milner J (2003). Disruption of the nucleolus mediates stabilization of p53 in response to DNA damage and other stresses. Embo j.

[5] Olson MO, Hingorani K, Szebeni A (2002). Conventional and nonconventional roles of the nucleolus. Int Rev Cytol.

[6] Olson MO (2004). Sensing cellular stress: Another new function for the nucleolus?. Sci STKE 2004.

[7] Montanaro L, Trere D, Derenzini M (2008). Nucleolus, ribosomes, and cancer. Am J Pathol.

[8] Levy DS, Kahana JA, Kumar R (2009). AKT inhibitor, GSK690693, induces growth inhibition and apoptosis in acute lymphoblastic leukemia cell lines. Blood.

[9] Nguyen le XT, Mitchell BS (2013). Akt activation enhances ribosomal RNA synthesis through casein kinase II and TIF-IA. Proc Natl Acad Sci U S A.

[10] Arabi A, Wu S, Ridderstrale K, Bierhoff H, Shiue C, Fatyol K, Fahlen S, Hydbring P, Soderberg O, Grummt I, Larsson LG, Wright AP (2005). c-myc associates with ribosomal DNA and activates RNA polymerase I transcription. Nat Cell Biol.

[11] Grandori C, Gomez-Roman N, Felton-Edkins ZA, Ngouenet C, Galloway DA, Eisenman RN, White RJ (2005). c-myc binds to human ribosomal DNA and stimulates transcription of rRNA genes by RNA polymerase I. Nat Cell Biol.

[12] Vita M, Henriksson M (2006). The myc oncoprotein as a therapeutic target for human cancer. Semin Cancer Biol.

[13] Bakshi R, Zaidi SK, Pande S, Hassan MQ, Young DW, Montecino M, Lian JB, van Wijnen AJ, Stein JL, Stein GS (2008). The leukemogenic t(8, 21) fusion protein AML1-ETO controls rRNA genes and associates with nucleolar-organizing regions at mitotic chromosomes. J Cell Sci.

[14] Drygin D, Lin A, Bliesath J, Ho CB, O'Brien SE, Proffitt C, Omori M, Haddach M, Schwaebe MK, Siddiqui-Jain A, Streiner N, Quin JE, Sanij E (2011). Targeting RNA polymerase I with an oral small molecule CX-5461 inhibits ribosomal RNA synthesis and solid tumor growth. Cancer Res.

[15] Peltonen K, Colis L, Liu H, Trivedi R, Moubarek MS, Moore HM, Bai B, Rudek MA, Bieberich CJ, Laiho M (2014). A targeting modality for destruction of RNA polymerase I that possesses anticancer activity. Cancer Cell.

[16] Peltonen K, Colis L, Liu H, Jaamaa S, Moore HM, Enback J, Laakkonen P, Vaahtokari A, Jones RJ, af Hallstrom TM, Laiho M (2010). Identification of novel p53 pathway activating small-molecule compounds reveals unexpected similarities with known therapeutic agents. PLoS One.

[17] Colis L, Peltonen K, Sirajuddin P, Liu H, Sanders S, Ernst G, Barrow JC, Laiho M (2014). DNA intercalator BMH-21 inhibits RNA polymerase I independent of DNA damage response. Oncotarget.

[18] Bywater MJ, Poortinga G, Sanij E, Hein N, Peck A, Cullinane C, Wall M, Cluse L, Drygin D, Anderes K, Huser N, Proffitt C, Bliesath J (2012). Inhibition of RNA polymerase I as a therapeutic strategy to promote cancer-specific activation of p53. Cancer Cell.

[19] Yuan X, Zhou Y, Casanova E, Chai M, Kiss E, Grone HJ, Schutz G, Grummt I (2005). Genetic inactivation of the transcription factor TIF-IA leads to nucleolar disruption, cell cycle arrest, and p53-mediated apoptosis. Mol Cell.

[20] Deisenroth C, Zhang Y (2010). Ribosome biogenesis surveillance: Probing the ribosomal protein-Mdm2-p53 pathway. Oncogene.

[21] Tapia C, Kutzner H, Mentzel T, Savic S, Baumhoer D, Glatz K (2006). Two mitosis-specific antibodies, MPM-2 and phospho-histone H3 (Ser28), allow rapid and precise determination of mitotic activity. Am J Surg Pathol.

[22] Jackson SP, Bartek J (2009). The DNA-damage response in human biology and disease. Nature.

[23] Sarkaria JN, Busby EC, Tibbetts RS, Roos P, Taya Y, Karnitz LM, Abraham RT (1999). Inhibition of ATM and ATR kinase activities by the radiosensitizing agent, caffeine. Cancer Res.

[24] Golding SE, Rosenberg E, Valerie N, Hussaini I, Frigerio M, Cockcroft XF, Chong WY, Hummersone M, Rigoreau L, Menear KA, O'Connor MJ, Povirk LF, van Meter T (2009). Improved ATM kinase inhibitor KU-60019 radiosensitizes glioma cells, compromises insulin, AKT and ERK prosurvival signaling, and inhibits migration and invasion. Mol Cancer Ther.

[25] Fokas E, Prevo R, Pollard JR, Reaper PM, Charlton PA, Cornelissen B, Vallis KA, Hammond EM, Olcina MM, Gillies McKenna W, Muschel RJ, Brunner TB (2012). Targeting ATR *in vivo* using the novel inhibitor VE-822 results in selective sensitization of pancreatic tumors to radiation. Cell Death Dis.

[26] Burger K, Muhl B, Harasim T, Rohrmoser M, Malamoussi A, Orban M, Kellner M, Gruber-Eber A, Kremmer E, Holzel M, Eick D (2010). Chemotherapeutic drugs inhibit ribosome biogenesis at various levels. J Biol Chem.

[27] Donati G, Brighenti E, Vici M, Mazzini G, Trere D, Montanaro L, Derenzini M (2011). Selective inhibition of rRNA transcription downregulates E2F-1: A new p53-independent mechanism linking cell growth to cell proliferation. J Cell Sci.

[28] Emmott E, Hiscox JA (2009). Nucleolar targeting: The hub of the matter. EMBO Rep.

[29] Audas TE, Jacob MD, Lee S (2012). Immobilization of proteins in the nucleolus by ribosomal intergenic spacer noncoding RNA. Mol Cell.

[30] Klein J, Grummt I (1999). Cell cycle-dependent regulation of RNA polymerase I transcription: The nucleolar transcription factor UBF is inactive in mitosis and early G1. Proc Natl Acad Sci U S A.

[31] Hernandez-Verdun D (2011). Assembly and disassembly of the nucleolus during the cell cycle. Nucleus.

[32] Wu MH, Yung BY (1994). Cell cycle phase-dependent cytotoxicity of actinomycin D in HeLa cells. Eur J Pharmacol.

