# Supplementary material for: rRNA synthesis inhibitor, CX-5461, activates ATM/ATR pathway in acute lymphoblastic leukemia, arrests cells in G2 phase and induces apoptosis
Source: Oncotarget. 2015 Jun 5;6(20):18094–104. doi: 10.18632/oncotarget.4093 (PMC4627237; doi:10.18632/oncotarget.4093)
Supplement: Supplementary file 1 [file oncotarget-06-18094-s001.pdf]

SUPPLEMENTARY FIGURES AND TABLES

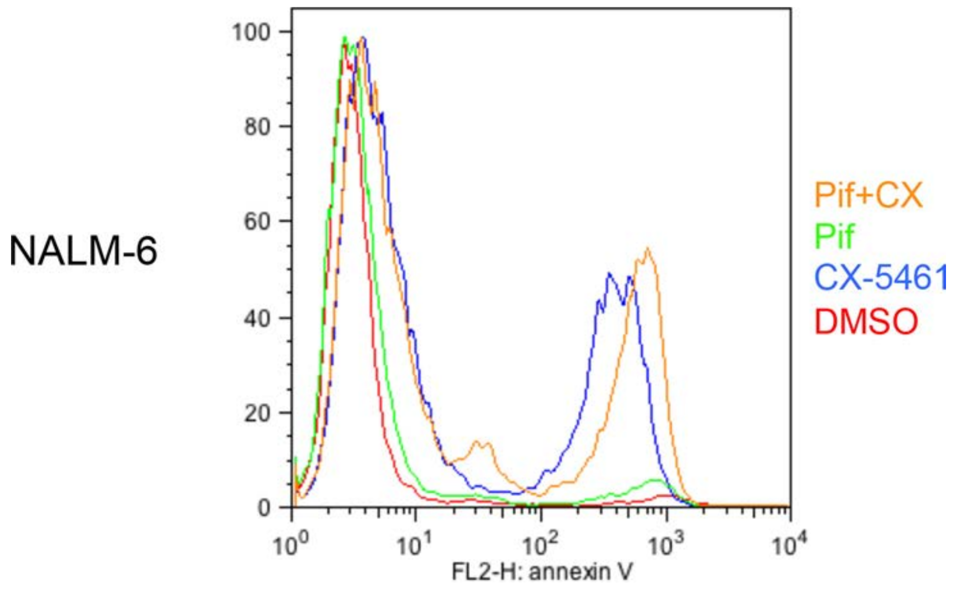

Supplementary Figure S1: p53 wild type NALM-6 cells were treated with 0.25 µM CX-5461 or 30 µM Pifithrin-α or both. Annexin V was used to measure apoptosis after 2 days. One representative experiment out of two is shown.

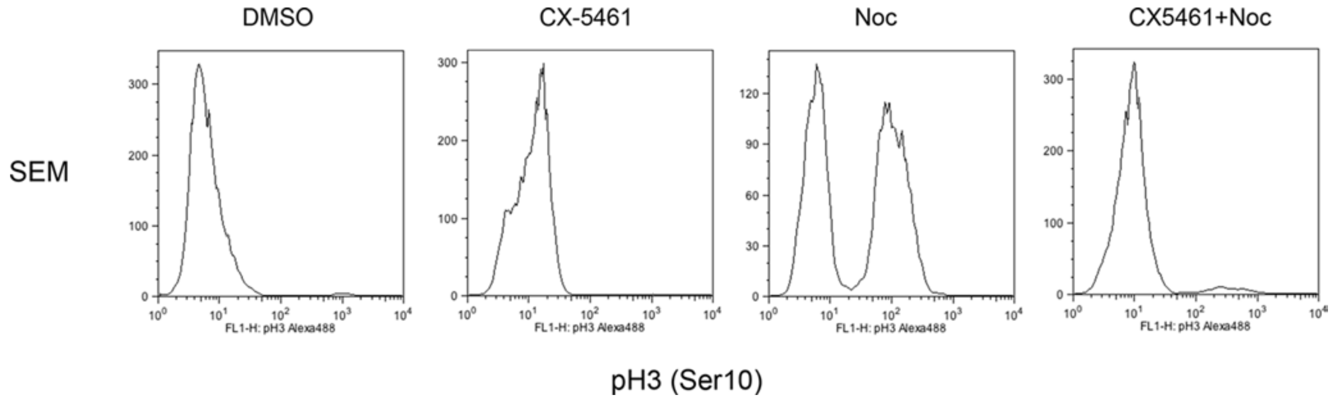

Supplementary Figure S2: SEM cells were treated with CX-5461, Nocodazole or 2 h pre-treatment with CX-5461 followed by nocodazole. Mitosis specific pH3(S10) antibody was used for flow cytometry.

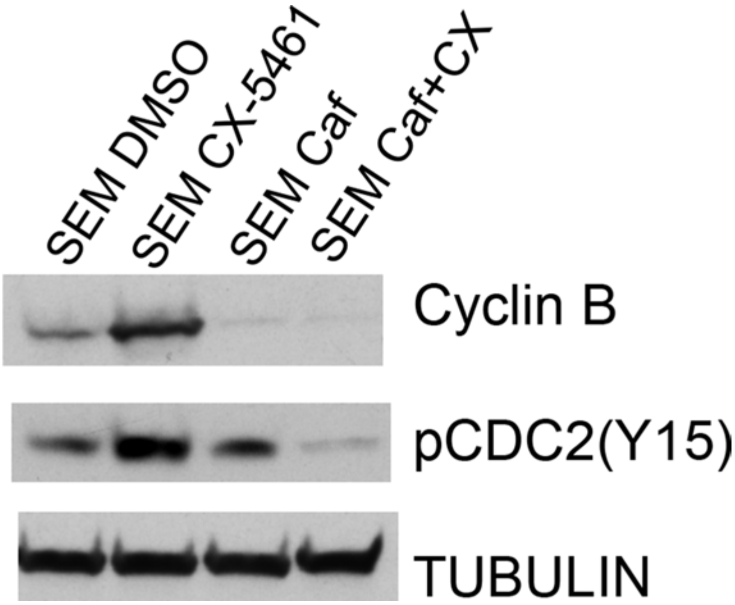

Supplementary Figure S3: Western blot was used to measure the levels of cyclin B, pCDC2(Y15) and tubulin in SEM cells treated with 0.25 µM CX-5461 for 1 day.

Supplementary Table 1. Cell Line

Cell Line	Translocation	p53 status if known
HB11;19	MLL-ENL	
REH	TEL-AML1	Mut
RS4;11	MLL-AF4	WT
SEM	MLL-AF4	Mut
KOPN-8	8 MLL-ENL	Mut
NALM-6	t(5;12)(q33.2;p13.2)	WT
697	t(1;19)(q23;p13), del(6)(q21)	WT
MUTZ-5	t(12;13)(p12;q13-14)	

Supplementary Table 2. Patient Samples

Patient Sample	Cytogenetics
P1	TEL-AML
P2	MLL-AF4
P3	Normal Karyotype
P4	MLL-ENL
P5	TEL-AML
P6	MLL-ENL
